# Phenotypic and genotypic characterization of clinical carbapenem-resistant *Acinetobacter* species harboring the metallo-beta-lactamases IMP-8 or NDM-1 in China

**DOI:** 10.1128/spectrum.01158-24

**Published:** 2024-12-27

**Authors:** Qingye Xu, Xinli Mu, Jintao He, Haiyang Liu, Xiaochen Liu, Yanfei Wang, Xiaoting Hua, Yunsong Yu

**Affiliations:** 1 Zhejiang Provincial People’s Hospital, People’s Hospital of Hangzhou Medical College, Hangzhou, Zhejiang, China; 2 Department of Infectious Diseases, Sir Run Run Shaw Hospital, Zhejiang University School of Medicine, Hangzhou, Zhejiang, China; 3 Key Laboratory of Microbial Technology and Bioinformatics of Zhejiang Province, Hangzhou, Zhejiang, China; 4 Regional Medical Center for National Institute of Respiratory Diseases, Sir Run Run Shaw Hospital, School of Medicine, Zhejiang University, Hangzhou, Zhejiang, China; 5 Centre of Laboratory Medicine, Zhejiang Provincial People’s Hospital, People’s Hospital of Hangzhou Medical College, Hangzhou, Zhejiang, China; Michigan State University, East Lansing, Michigan, USA

**Keywords:** MBLs, Class 1 integron, ST150, p*dif*

## Abstract

Carbapenem-resistant *Acinetobacter* spp. pose a significant challenge in clinical settings due to limited treatment options for nosocomial infections. Carbapenem-hydrolyzing class D beta-lactamases are the primary cause for carbapenem resistance, while metallo-beta-lactamases (MBLs) New Delhi metallo beta-lactamase (NDM) and imipenemase (IMP) also contribute. This study investigated five MBL-producing *Acinetobacter* spp. strains isolated from a clinic in China in 2010. The *bla*_IMP-8_-carrying A1014 was the first identified CRAB among all known ST_Pas_150 isolates worldwide. Through whole-genome sequencing and the southern blot analysis, we determined that *bla*_IMP-8_ was located on a pR4WN-type plasmid and *bla*_NDM-1_ was located on four distinct pSU1904NDM-type plasmids. The *bla*_IMP-8_ gene was identified within a class 1 integron organized as a 5’-conserved segment (*intI*), variable region (*bla*_IMP-8_-*aac(6')-Ib*), and 3’-conserved segment (*qacE*Δ1/*sul1*). All available sequences of *bla*_IMP_ variants in *A. baumannii* from the NCBI were investigated and classified into five types of class 1 integrons. All *bla*_NDM-1_ plasmids were transferable, and the *bla*_NDM-1_ genes were in a conservative region. Additionally, multiple resistance genes, including those conferring resistance to aminoglycosides, tetracyclines, and macrolides, were detected on plasmids from these strains. All strains were resistant to meropenem and imipenem, while they were all susceptible to tigecycline and intermediate to polymyxin. A207 and A1014 were susceptible to cefiderocol, and only *bla*_IMP-8_-carrying A1014 had low MIC value (4/2 µg/mL) toward cefoperazone/sulbactam. In conclusion, we characterized the phenotypic and genotypic features of one IMP-8-producing and four NDM-1-producing plasmids recovered from *Acinetobacter* spp. strains isolated in 2010, contributing to the understanding of the dissemination and evolution of these enzymes.

**IMPORTANCE:**

Given the low prevalence of IMP among *A. baumannii* and the limited sequencing technology in earlier years, research on *bla*_IMP_ in *A. baumannii* is scarce, and genetic information on *bla*_NDM-1_-producing *Acinetobacter* spp. strains isolated in earlier years is limited. This study revisited five MBL-carrying *Acinetobacter* spp. strains isolated in 2010, characterizing their phenotypic and genotypic features. This retrospective analysis serves as a form of “bacterial archaeology,” providing evidence of the evolutionary changes in genetic elements conferring antibiotic resistance.

## INTRODUCTION

Carbapenem-resistant *Acinetobacter* species have become a thorny issue for a long time, significantly limiting treatment options for nosocomial infections, particularly in the context of the COVID-19 pandemic (1, 2). The production of carbapenem-hydrolyzing class D beta-lactamases OXA is the most common mechanism of carbapenem resistance in *Acinetobacter* species. However, metallo-beta-lactamases (MBLs), like New Delhi metallo beta-lactamase (NDM) and imipenemase (IMP), are also prevalent in *Acinetobacter* across Asia (3–7).

Since the initial report of *bla*_IMP_ in Japan in 1991, this gene has been frequently transferred between organisms, with *Enterobacterales* and *Pseudomonas aeruginosa* being the most common carriers of IMP (8). To date, 96 variants have been identified worldwide, typically disseminating through class 1 or class 3 integrons, and 10 variants have been detected in *A. baumannii* (7). In China, *bla*_IMP_ was first detected in 23 strains from a Hong Kong hospital in the late 1990 s, with *bla*_IMP-4_ being a new variant at that time (4). Subsequently, *bla*_IMP-1_ and *bla*_IMP-8_ were successively detected in *Acinetobacter* in Taiwan and mainland China (9–12).

NDM is the most common MBL in *Acinetobacter*, first discovered in 2009 in a *Klebsiella pneumoniae* strain recovered from an India-origin Swedish patient who had traveled to New Delhi, India (13). Shortly thereafter, our nationwide survey conducted in 2009 and 2010 revealed the emergence of NDM-1 specifically within *Acinetobacter* species in China, not among other Gram-negative bacteria (5, 6). Until now, NDM is still a major class B β-lactamase in hospital settings, especially in intensive care units (ICUs) (14–17).

Over the decades, reports of MBLs in *Acinetobacter* have been frequent, but genetic analysis has been limited due to technological constraints, particularly at the time of their emergence. Here, in this study, we characterized the genetic and phenotypic features of MBL-producing plasmids recovered from the *Acinetobacter* spp. strains isolated in 2010. This work holds significant importance when examining the historical context of the dissemination and evolution of these enzymes.

## MATERIALS AND METHODS

### Bacterial isolation, Identification, and antimicrobial susceptibility testing

All strains in this study (Table 1) were isolated from patients in China in 2010. Species were determined by calculating average nucleotide identities (ANIs) using JSpeciesWS (https://jspecies.ribohost.com/jspeciesws/), with *Acinetobacter baumannii* ATCC 19606 and *Acinetobacter pittii* ATCC 19004 used as the type strains, respectively (18). Minimum inhibitory concentrations (MICs) of antimicrobial agents were determined by the broth microdilution method following the Clinical and Laboratory Standards Institute (CLSI) guidelines, and susceptibility results were also interpreted according to CLSI guidelines (19). *Escherichia coli* ATCC25922 was tested as the quality control. Since tigecycline susceptibility data did not have established breakpoints in the guidelines of either the CLSI or the European Committee on Antimicrobial Susceptibility Testing (EUCAST), the FDA-identified interpretive criteria for *Enterobacterales* were employed, with a minimum inhibitory concentration of ≤2 indicating susceptibility (20).

**TABLE 1:** Characteristics of five MBL-producing *Acinetobacter* spp. isolates

Strain	Year of isolation	Species	Origin	Specimen
A207	2010	*A. baumannii*	Urumqi Municipality, Xinjiang	Sputum
A774	2010	*A. pittii*	Hangzhou, Zhejiang	Urine
A1014	2010	*A. baumannii*	Lishui, Zhejiang	Sputum
A1269	2010	*A. pittii*	Haikou, Hainan	Sputum
XH1038	2010	*A. baumannii*	Hanzhong, Shaanxi	Secretions

### Whole-genome sequencing and genomic analysis

Genomic DNA was extracted using a Qiagen minikit according to the manufacturer’s instruction and was sequenced through Illumina HiSeq platform along with the MinION Nanopore. Unicycler v0.4.8 was used to assemble and polish the sequences (21). Prokka v1.13 was utilized for annotation (22). The sequence of XH1038 was obtained from our recent Bioproject PRJNA1016089 at the NCBI. MLST on the Center for Genomic Epidemiology website was used to scheme the sequence type. Comprehensive Antibiotic Resistance Database (CARD) was used to detect the acquired antimicrobial resistance genes (23, 24). The type IV secretion system (T4SS) region was predicted using oriTfinder with default parameter settings (25). p*dif* sites were identified via pdiffinder (26).

All sequences used for comparative analyses were downloaded from the NCBI database, as well as the 36 ST_Pas_150 *A. baumannii* strains used for constructing the phylogenetic tree with A1014. The phylogenetic tree of ST_Pas_150 isolates was built with Snippy v4.4.5 (https://github.com/tseemann/snippy) and FastTree using RAxML under the GTRGAMMA model with A1014 as the reference strain and then visualized with iTOL v5 (https://itol.embl.de) (27). Plasmid structures were visualized by Proksee (https://proksee.ca/projects). Sequence alignment of the plasmids was conducted by BRIG or Easyfig (28, 29). Results were further visualized with Adobe Illustrator 2020 manually.

### S1-PFGE and southern blot

To confirm the location of the *bla*_NDM-1_ and *bla*_IMP-8_, genomic DNA digested with S1-nuclease was electrophoresed on a CHEF-mapper XA pulsed-field gel electrophoresis (PFGE) system (Bio-Rad, USA) at 14°C for 20 hours with run conditions of 6 V/cm and pulse times from 2.16 seconds to 63.8 seconds. And the fragments in the gel were transferred to a nylon membrane (Millipore, USA) and then hybridized with digoxigenin-labeled *bla*_NDM-1_ and *bla*_IMP-8_ probe, respectively. The target genes were detected by the NBT/BCIP color detection kit.

### Conjugation and transformation

To investigate the transferability of the *bla*_NDM-1_ and *bla*_IMP-8_, filter mating was performed using a rifampicin-resistant derivative of *A. baumannii* ATCC17978 as the recipient strain (17). The donor and recipient were added at a ratio of 1:1 on a filter membrane placed on MH agar without antibiotics and incubated at 37°C overnight. Transconjugants were selected on Mueller–Hinton (MH) agar in the presence of rifampicin (50  µg/mL) and meropenem (4  µg/mL). Confirmation of the transconjugants was performed using PCR with primers targeting the *bla*_NDM-1_ or *bla*_IMP-8_ and the replicons of each plasmid, and primers specific to ATCC17978 were used to exclude donors (Table S1).

Conjugation rate (expressed as mL cell^−1^ h^−1^) and conjugation frequencies (the number of transconjugants per recipient) were conducted as previously described (30, 31). Specifically, the donor and recipient were separately incubated in MH broth overnight and then 5 uL of the donor and recipient were inoculated together into a tube containing 5 mL MH broth and incubated at 37°C with 200 rpm for 24 hours. OD_600_ of the mixed culture was measured at 3 and 4 hours post-incubation to calculate the maximum growth rate during the exponential phase. φ is the maximum growth rate of the mixed donor and recipient culture, which is calculated from OD_600_ measured at two time points (hour) during the exponential phase: $\varphi =\frac{ln({OD}_{b}/{OD}_{a})}{t_{b}-t_{a}}$. The overnight mixed culture was diluted and spread onto agar media. We calculated the number of colonies (CFU/mL) that could grow on antibiotic-free MH agar (N), 4 mg/L meropenem MH agar (A), 50 mg/L rifampicin MH agar (B), and 4 mg/L meropenem plus 50 mg/L rifampicin MH agar (T). The number of donors (D) = A T, and the number of recipients (R) = B. The cell densities were then used to calculate the conjugation rate λ (ml cell^−1^ h^−1^) as follows: $\lambda =\varphi ln(1+\frac{T}{R}∙\frac{N}{D})\frac{1}{N}$. The conjugation frequency was also calculated using T and R in the same culture. Each strain was tested three times.

Moreover, plasmid DNA of the strain A1014 was extracted with the Qiagen Midi Kit due to its failure in filter mating. Transformation of this plasmid was carried out using ATCC17978 as the recipient strain through electroporation, and MH agar containing meropenem (1 µg/mL) was used to screen the transformants.

## RESULT

### Characterization of five MBL-carrying *Acinetobacter* spp. strains

**Fig 1:**
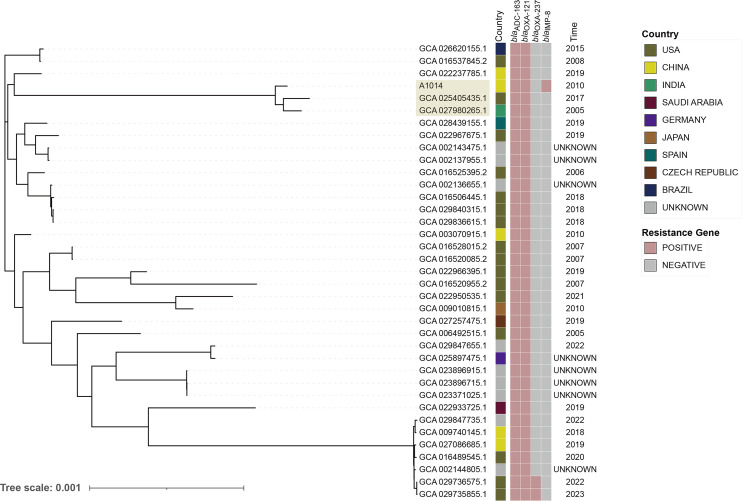
The phylogenetic tree of A1014 and other 36 ST_Pas_150 *A. baumannii* strains. The characteristics of each strain are shown on the right, including isolation geo location, β-lactamase resistance genes, and isolation time.

*A. baumannii* A1014, which carried *bla*_IMP-8_, was recovered from sputum samples in Zhejiang (Table 1). The genome of A1014 comprised a 3,844,255-bp chromosome and a 316,034-bp plasmid. The native *ampC* gene *bla*_ADC-163_ and *oxaAb* β-lactamase *bla*_OXA-121_ were encoded on the chromosome, while *bla*_IMP-8_ was located on the plasmid along with several other antimicrobial resistance genes (Table 2). A1014 exhibited resistance to meropenem and imipenem and was susceptible/intermediate to other agents tested (Table 3). According to the Oxford and Pasteur schemes, A1014 was identified as ST_Oxf_744 and ST_Pas_150, which is a rare sequence type. To investigate this rare IMP-producing isolate of a rare sequence type, we screened the accessible *A. baumannii* sequences from the NCBI database, a total of 36 ST_Pas_ 150 strains and 12 ST_Oxf_ 744 strains were obtained. Meanwhile, the entire 12 ST_Oxf_744 strains belonged to ST_Pas_150. Then, phylogenomic analysis was performed using the core genome of A1014 and these 36 strains. As shown in Fig. 1, these strains were isolated from diverse geographical locations since 2005, mainly in America and Asia. A1014 was found to cluster with two strains from India and the USA, spanning more than a decade. Notably, the beta-lactam resistance profiles of these ST_Pas_150 strains were relatively straightforward, with all strains harboring the intrinsic *bla*_OXA-121_ and *bla*_ADC-163_ β-lactamase genes. Only two strains additionally encoded the carbapenem-hydrolyzing class D beta-lactamase OXA-237. In these two strains, IS*Aba1* was detected upstream of the *bla*_OXA-237_, while their susceptibility profile to carbapenems has not been confirmed. Additionally, no IS*Aba1* or other insertion sequences were detected upstream of the intrinsic *bla*_OXA-121_ and *bla*_ADC-163_ genes in these strains. A1014, carrying the metallo-beta-lactamase IMP-8, was unequivocally identified as carbapenem-resistant among all these ST_Pas_150 strains.

**TABLE 2:** Genetic characteristics of five MBL-carrying isolates

Strain	^*a*^ST_Oxf_	^*b*^ST_Pas_	Chromosome/plasmid	Size (bp)	G + C content (mol%)	Replicon	Antimicrobial resistance gene	Accession number
A207	2220	152	Chromosome	3,749,633	39.20		*bla*_OXA-120_, *bla*_ADC-25_	CP132915
			Plasmid: pA207-1	108,969	40.99	GR24		CP132916
			Plasmid: pA207-2	50,328	40.56	pSU1904NDM	*aph(3')-VI*, *bla*_NDM-1_	CP132917
			Plasmid: pA207-3	11,779	37.13	GR26		CP132918
			Plasmid: pA207-4	11,086	36.00	Aci7		CP132919
A774			Chromosome	3,986,977	39.13		*bla*_OXA-417_, *bla*_ADC-25_	CP133054
			Plasmid: pA774-1	149,365	40.79	GR24		CP133055
			Plasmid: pA774-2	47,271	40.78	pSU1904NDM	*aph(3')-VI*, *bla*_NDM-1_	CP133056
			Plasmid: pA774-3	13,064	35.88	GR18		CP133057
			Plasmid: pA774-4	11,959	33.62	repA1343		CP133058
			Plasmid: pA774-5	7,793	35.94	Aci2b		CP133059
A1014	744	150	Chromosome	3,844,255	39.04		*bla*_OXA-121_ and *bla*_ADC-163_	CP133052
			Plasmid: pA1014-1	316,034	38.33	pR4WN	*msr(E*), *mph(E*), *aac(6')-Ib*, *aac (3)-IId*, *sul1*, *sul2*, *tet (39*), and *bla*_IMP-8_	CP133053
A1269			Chromosome	3,759,218	38.92		*bla*_OXA-1045_ and *bla*_ADC-25_	CP133047
			Plasmid: pA1269-1	43,578	39.16	GR22	*msr(E*), *mph(E*), *aac(3'')-Ib*, *aph (6)-Id*, *sul2*, and *tet (39*)	CP133048
			Plasmid: pA1269-2	39,365	37.26	pSU1904NDM	*aac(3')-VI* and *bla*_NDM-1_	CP133049
			Plasmid: pA1269-3	6,974	33.45	repA1343		CP133050
			Plasmid: pA1269-4	4,740	36.12	^*c*^ND		CP133051
XH1038	867	239	Chromosome	3,723,049	38.99		*bla*_OXA-51_ and *bla*_ADC-25_	CP134566
			Plasmid: pXH1038-1	47,274	40.82	pSU1904NDM	*aph(3')-VI* and *bla*_NDM-1_	CP134567
			Plasmid: pXH1038-2	12,471	35.43	repA1343		CP134568
			Plasmid: pXH1038-3	5,936	36.67	GR1		CP134569

^*a*^ ST_Oxf_, typing according to the Oxford MLST scheme.

^*b*^ ST_Pas_, typing according to the Pasteur MLST scheme.

^*c*^ ND, not detected.

**TABLE 3:** Minimum inhibitory concentrations of antimicrobial agents

Strain	Minimum inhibitory concentration (µg/mL)							
	Cefoperazone/sulbactam	Polymyxin	Tigecycline	Amikacin	Ciprofloxacin	Meropenem	Imipenem	Cefiderocol
A207	128/64	2	0.125	16	0.25	128	64	4
A774	128/64	1	0.125	32	0.125	>128	128	16
A1014	4/2	2	0.125	4	0.125	8	32	1
A1269	>128/64	1	0.25	32	>32	>128	128	32
XH1038	>128/64	2	0.125	16	0.25	>128	128	32
ATCC17978Rif^R^	4/2	1	0.25	1	0.25	0.5	0.125	0.25
T^*a*^-A207	>128/64	1	0.125	4	0.125	>128	128	2
T-A774	>128/64	1	0.25	8	0.25	>128	128	2
T-A1269	>128/64	1	0.125	4	0.125	>128	64	2
T-XH1038	>128/64	1	0.25	4	0.25	>128	128	2

^*a*^ T, ATCC17978 transconjugants of A207, A774, A1269, and XH1038.

^*b*^ Grey shade represents recipient strain and transconjugants.

Four *Acinetobacter* spp. strains carried *bla*_NDM-1_, of which *A. baumannii* A207 (ST_Pas_152) and XH1038 (ST_Pas_239) were isolated from sputum in Xinjiang and Zhejiang, and *A. pittii* strains A774 and A1269 were isolated from urine and secretions from Zhejiang and Shaanxi, respectively (Table 1). The *bla*_ADC-25_ and intrinsic *bla*_OXA-51_/*bla*_OXA-213_-like genes were present in each *A. baumannii* and *A. pittii* chromosome. Four *bla*_NDM-1_ genes were all located on pSU1904NDM-type plasmids, which additionally co-harbored an aminoglycoside resistance gene *aph(3')-VI*, despite variations in plasmid size. Beyond that, *A. pittii* A1269 held another 43,578-bp GR22-type plasmid pA1269-1 with resistance genes *msr(E*), *mph(E*), *aac(3'')-Ib*, *aph (6)-Id*, *sul2,* and *tet*(39) (Table 2). This plasmid with multidrug resistance genes displayed extreme similarity with those of another two plasmids pAP2044-2 (CP087718.1) and pAP8900-2 (CP123767.1) (Fig. S1). AP2044 and AP8900 were both *A. pittii* strains and collected in China, while AP2044 was isolated from hydrothorax in Luzhou in 2020, and AP8900 was isolated from sputum in Zhuhai in 2022.

These four strains showed high MIC values toward cefoperazone/sulbactam, imipenem, and meropenem and were susceptible or intermediate to amikacin. However, A1269 was resistant to ciprofloxacin (MIC >32 mg/L) due to the Ser81Leu in *gyrA*. All strains demonstrated susceptibility to tigecycline, with MIC values ranging from 0.125 to 0.25 µg/mL. Regarding polymyxin, all strains showed intermediate resistance, with MIC values ranging from 1 to 2 µg/mL, which falls within the intermediate category ≤2 µg/mL, as defined by CLSI guidelines. As for cefiderocol, three isolates exhibited resistance, while A207 remained susceptible (Table 3).

### Genetic characterization of the *bla*_IMP-8_–carrying plasmid in *A. baumannii* A1014

**Fig 2:**
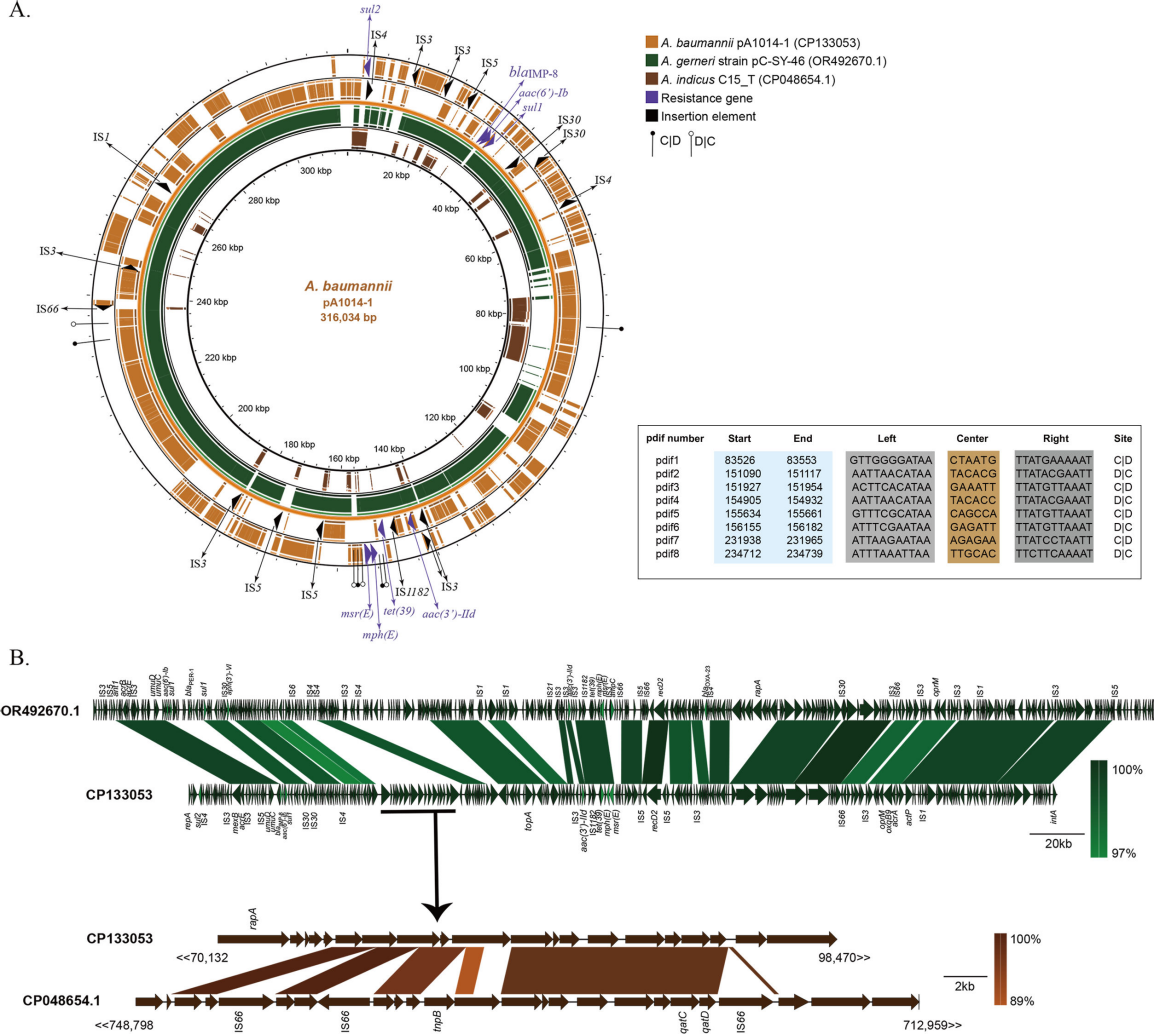
Circular map and genetic environment of the pA1014-1 plasmid. (**A**) Circular map of the pA1014-1 plasmid and the comparison with plasmid pC-SY-46 (OR492670.1) and C15_T chromosome (CP048654.1). The height of the alignment arc represented the query identity. Resistance genes and insertion elements were shown in purple and black regions, respectively, and the names were labeled with arrows. p*dif* sites were All tagged by solid and hollow marks. All the 28-bp p*dif* sites are listed below, 6-bp sequences are shown in the central region, and the 11-bp sequences were labeled as left and right arms. (**B**) Comparison between plasmid pC-SY-46 (OR492670.1), pA1014-1 (CP133053), and C15_T chromosome (CP048654.1) using Easyfig. Resistance genes are labeled with light green.

The whole-genome sequencing revealed that the *bla*_IMP-8_–carrying plasmid pA1014-1 was a pR4WN-type huge plasmid, with a GC content of 38.33% on average. S1-PFGE and southern blot also confirmed that the *bla*_IMP-8_ was on a plasmid between 310.1 and 336.5 Kbp in size (Fig. S2). In addition to *bla*_IMP-8_, pA1014-1 contained other resistance genes conferring resistance to macrolide [*msr(E*) and *mph(E*)], aminoglycosides [*aac(6')-Ib*, *aac (3)-IId*], sulfonamide (*sul1* and *sul2*), and tetracycline [*tet (39*)]. Moreover, eight p*dif* sites were found in the pA1014-1 plasmid, with *msr(E)-mph(E*) located in the *dif* module(Fig. 2A).

pA1014-1 shared good genetic similarity with pC-SY-46 (OR492670.1; 83% coverage and 99.39% identity), a plasmid recovered from *A. gerneri* C-SY-46 from sewage water in Hangzhou, China, in 2023. Nevertheless, the *bla*_IMP-8_ gene was lacked in the integron of pC-SY-46, and pC-SY-46 exhibited poor similarity to the 70–100 Kbp region in pA1014-1 (Fig. 2A). The chromosome of the *A. indicus* strain C15_T (CP048654.1) was found highest, similar with the 70–100 Kbp region through BLAST at the NCBI database, showing 56% coverage and 97.82% identity (Fig. 2). This *A. indicus* C15_T was also isolated in China in 2015, yet isolated from animal feces.

**Fig 3:**
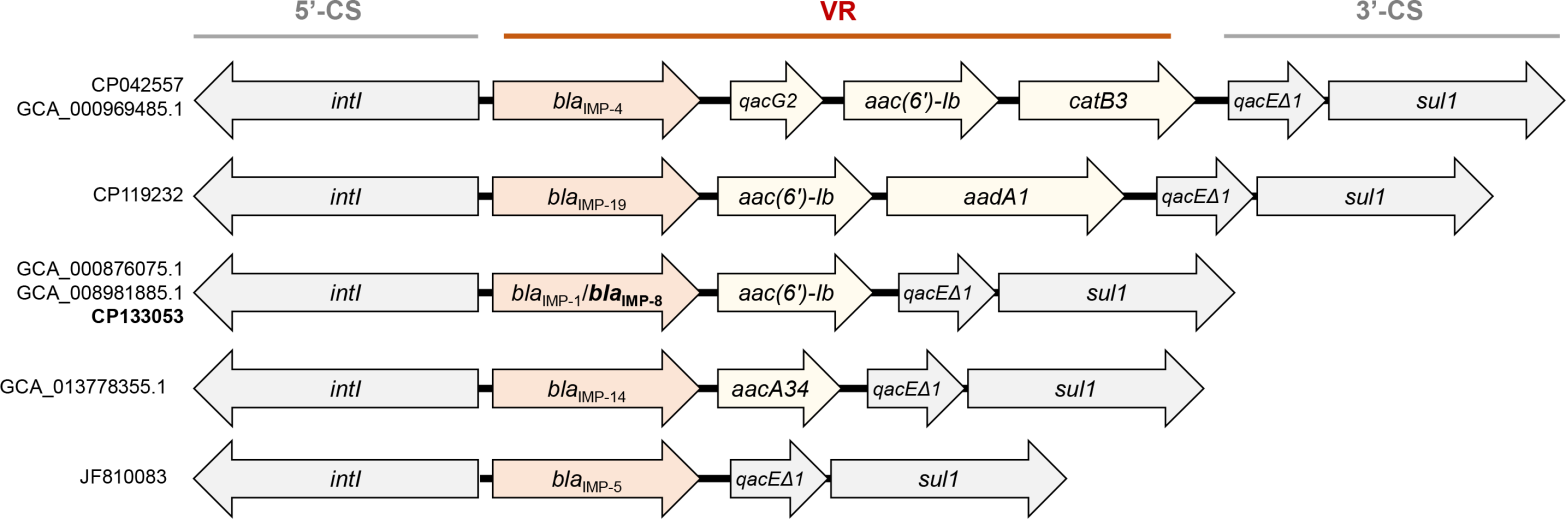
Schematic representation of different class 1 integrons carrying the *bla*_IMP_ gene in *A. baumannii*. Arrows indicated transcription directions of genes. 5′-CS and 3′-CS are in gray. The variable region is labeled with yellow, and *bla*_IMP_ is highlighted. The figure is not to scale.

The *bla*_IMP-8_ gene in pA1014-1 was encoded in a class 1 integron organized as a 5’-conserved segment (5′-CS) (*intI*), variable region (VR) (*bla*_IMP-8_-*aac(6')-Ib*), and 3′-CS (*qacE*Δ1/*sul1*). All available sequences of *bla*_IMP_-positive *A. baumannii* at NCBI were downloaded to further investigate the gene context of *bla*_IMP_ in *A. baumannii*; these integrons shared the same basic platform, but there were five different VR components (Fig. 3). The schematic plot contained six *bla*_IMP_ genotypes: *bla*_IMP-1,_*bla*_IMP-4,_*bla*_IMP-5,_*bla*_IMP-8_, *bla*_IMP-14,_ and *bla*_IMP-19_, and the *bla*_IMP-8_ and *bla*_IMP-1_ shared the same VR structure. The variable regions of other incomplete integrons of *bla*_IMP_-positive *A. baumannii* strains downloaded from NCBI are also listed in Fig. S3. Among all the different contexts, aminoglycoside acetyltransferase genes were most commonly co-harbored with *bla*_IMP_, followed by other β-lactamases.

### Genetic characterization of four *bla*_NDM-1_–carrying plasmids

**Fig 4:**
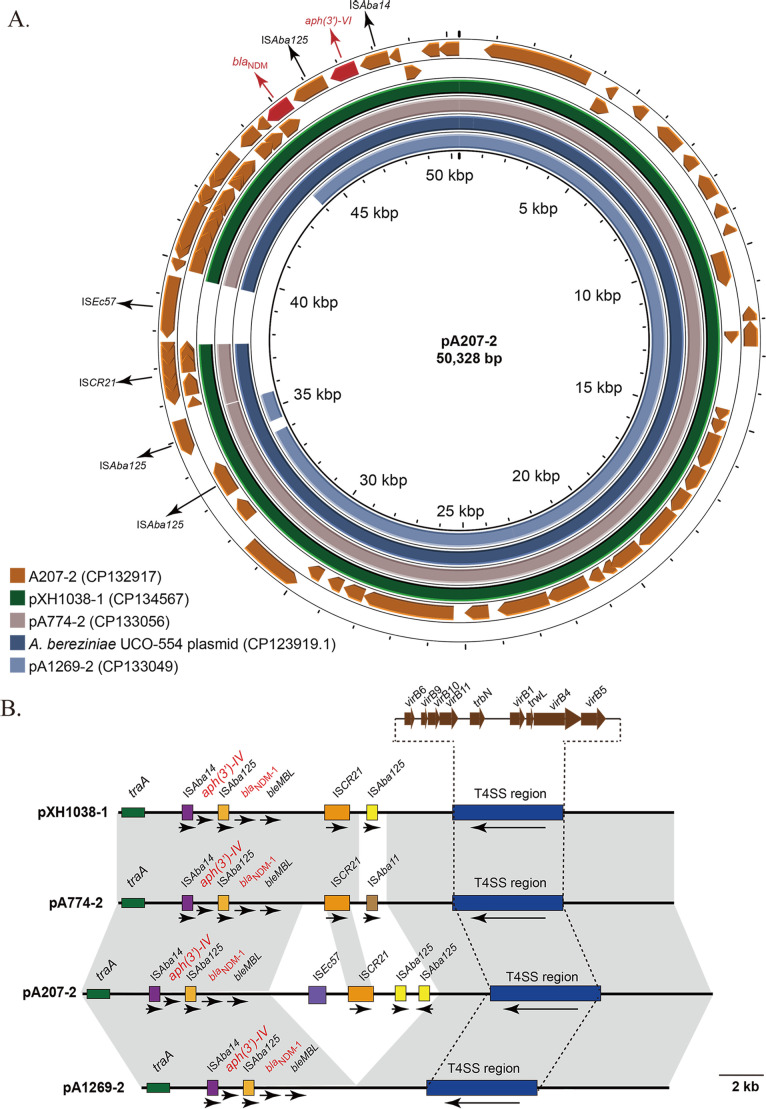
Plasmid comparison with *bla*_NDM-1_-harboring plasmids. (**A**) Circular map of *bla*_NDM-1_-harboring plasmid pA207-2(CP132917) and the comparison of the four *bla*_NDM-1_-harboring plasmids in this study with the UCO-554 plasmid (CP123919.1). Orange arrows represent its ORFs, red arrows and labels represent resistance genes, and black ones represent insertion elements. (**B**) Comparison of four *bla*_NDM-1_-harboring plasmids in this study. Resistance genes were labeled as red name, with the black arrows indicating the direction. Blue filled boxes indicate the T4SS region. Yellow filled boxes represent the IS*Aba125*. Shades indicate regions with identity of 99.99% to 100%.

Consistent with the S1-PFGE and southern blot, the sizes of these four *bla*_NDM-1_–carrying plasmids were less than 54.7 Kbp, with pA1269-2 being the smallest. The sequence of the largest plasmid, pA207-2 (CP132917), was used as the reference. As shown in Fig. 4A, the four plasmids shared high similarity. pXH1038-1 and pA774-2 plasmids shared high similarity (99% coverage and 99.99% identity). Moreover, 97% sequence coverage and 100% identity were identified between pA774-2 and pA207-2. Regarding pA207-2 and pA1269-2 plasmids, only 82% coverage was observed, but with 100% identity. In addition, when searching for similar sequences of pA207-2 using BLAST analysis at the NCBI, dozens of sequences were found having significant alignments. Among them, *Acinetobacter* spp. consumed a large proportion. The most similar one, plasmid (CP123919.1) from *A. bereziniae* UCO-554, recovered from patient blood in Chile in 2020, had 96% coverage and 100.00% identity with pA207-2. The plasmid of UCO-554 not only exhibited identical size to pXH1038-1 but also demonstrated complete coverage (100%) and a remarkably high sequence identity of 99.98% with pXH1038-1.

As for the genetic context of *bla*_NDM-1_, IS*Aba14-aph(3’)-VI*-IS*Aba125-bla*_NDM-1_-*ble*_MBL_-*iso-dsbD-cutA-groES-groL*-IS*CR21* was the most common structure among these four plasmids. The Tn*125* transposon made up of two copies of IS*Aba125* was identified in pXH1038-1 and pA207-2, while IS*Aba11* replaces the downstream IS*Aba125* in pA774-2 and the downstream IS*Aba125* was absent in pA1269-2 (Fig. 4B). Furthermore, each plasmid encoded a T4SS region composed of *virB6-virB9-virB10-virB11-trbN-virB1-trwL-virB4-virB5*.

### Transferability of the *bla*_NDM-1_ and *bla*_IMP-8_ plasmids

All the four NDM-carrying plasmids were successfully transferred to the rifampicin-resistant ATCC17978 through filter mating, indicating that the plasmids pA774-2 and pA1269-2 could be transmitted by interspecies lateral transfer. We further performed conjugation assay in liquid medium; except for the conjugation frequency (transconjugants per recipient) of A1014, which was below the limit of detection (<1.76 × 10^−6^), the frequency of other four *bla*_NDM-1_-carrying plasmids ranged from 7.14 × 10^−6^ to 2.13 × 10^−3^ (Fig. S4). Additionally, the conjugation rate was consistent with conjugation frequency, ranging from 1.06 × 10^−15^ to 7.03 × 10^−13^ ml cell^−1^ h^−1^, representing the number of transconjugants per density per hour (Fig. S4).

All transconjugants showed high MIC values toward cefoperazone/sulbactam, meropenem, and imipenem (Table 3). Moreover, compared to the recipient rifampicin-resistant ATCC17978, all transconjugants displayed higher MICs to amikacin and cefiderocol but still remained susceptible.

However, despite numerous attempts, the *bla*_IMP-8_-carrying plasmid in A1014 could not be transferred through conjugation nor transformation. This demonstrated that the *bla*_IMP-8_-carrying plasmid pA1014-1 cannot be readily transferred.

## DISCUSSION

The ability of *Acinetobacter* spp. to acquire resistance genes poses a great threat to clinical settings. Carbapenem-hydrolyzing class D beta-lactamases account for almost all carbapenem-resistant *Acinetobacter*, with *bla*_OXA_ being the most popular, followed by MBLs (32). In this study, we characterized the phenotypic and genetic features of one IMP-8-producing plasmid and four NDM-1 plasmids recovered from the *Acinetobacter* spp. strains isolated in 2010.

Due to the low prevalence of IMP among *A. baumannii* and the underdeveloped sequencing technology in earlier years, studies on *bla*_IMP_ in *A. baumannii* are limited, and only five types of *bla*_IMP_-carrying integrons were found when we screened the accessible sequence from NCBI (7, 33). Class 1 integrons play a crucial role in spreading antibiotic resistance among bacteria in the clinic. The mobility of gene cassettes in Class 1 integron, which probably are the antibiotic resistance genes, is mediated by the IntI1 (34). *bla*_IMP_ genes are often located in Class 1 accessory genetic elements harbored on broad-host-range plasmids or chromosomal accessory genetic elements (35). Therefore, there is a possibility that integrons help transmit this gene to other species. To our knowledge, *bla*_IMP-8_ was first reported in *Acinetobacter* in 2007 in China, and during the same period, the *bla*_IMP-8_ strain was also epidemic in *Enterobacteriaceae* and *A. baumannii* in Taiwan, China (10–12, 36). According to the isolation time and region, it seemed likely that IMP-8-producing *Acinetobacter* was once prevalent on China’s southeast coast. However, either loss of strains or lack of sequencing precludes retrospective studies. Macesic’s work, which analyzed clinical and environmental isolates from a single hospital over two decades and provided a framework for understanding the endemicity of IMP-4-producing organisms, including prevalence, transmission dynamics, and genetic characterization within that hospital, impressed us (37). Their work, to a large extent, encouraged us to conduct this retrospective analysis and convinced us that our complement for the past will show its value in the future.

The *bla*_IMP-8_-carrying *A. baumannii* A1014 belongs to a rare ST, with only other 36 ST_Pas_150 strains screened from the NCBI, most of which only harbored intrinsic *bla*_ADC-163_ and *bla*_OXA-121_ β-lactamase genes. Among these ST_Pas_150 strains, only A1014 was definitely carbapenem-resistant. The backbone of the IMP-8-producing plasmid was blasted to a plasmid recovered from *A. gerneri* C-SY-46 (OR492670.1), and our pA1014-1 was slightly smaller. This *A. gerneri* was isolated from hospital sewages in Zhejiang in 2023, implying such plasmids might have existed and evolved for over a decade. Also, the region that was not aligned to pC-SY-46 was similar to the chromosome of *A. indicus* strain C15_T (CP048654.1), recovered from pig feces in Guangdong in 2015 (38). Compared to pA1014-1, C15_T had multiple IS*66* insertions in that region, indicating that several recombination events occurred during the period of becoming part of the chromosome. The comparative analyses showed that there is a possibility of inter-species gene exchange in *Acinetobacter* species. Nevertheless, *bla*_IMP-8_ remains relatively uncommon compared to *bla*_IMP-1_ and *bla*_IMP-4_, with only few reports in *Acinetobacter* and *Enterobacterales*, primarily isolated from China (7). We suggest that the low detection rate of IMP-8 could be attributed to two primary factors: first, strains producing IMP-8 may potentially exhibit sensitivity to carbapenems or exhibit negative results in enzyme typing assays, which could lead to underreporting of IMP-8 in clinical and surveillance studies (39). Second, the integrons or plasmids harboring *bla*_IMP-8_ may possess limited transferability, which would restrict the dissemination of this gene.

In contrast, *bla*_NDM-1_ can be easily horizontally transferred through conjugation or outer membrane vesicles (40, 41). Therefore, the *bla*_NDM-1_ plasmids in *A. baumannii* are more common than *bla*_IMP-8_ and relatively conservative during dissemination. Moreover, *bla*_NDM-1_-carrying plasmids have the potential for interspecies lateral transfer, and the plasmid (CP123919.1) from the currently isolated *A. bereziniae* UCO-554 demonstrated a striking resemblance to the plasmids investigated in this study. Dozens of sequences of *Acinetobacter* spp. similar to pA207-2 were found at the NCBI database. Despite a decade-long difference in their isolation times, the observed similarities suggest an enduring presence and conservation of these plasmids over time.

Although the *bla*_NDM-1_ transconjugants were susceptible to cefiderocol, their donors were more likely to be resistant to this new drug, which might arise from the existence of other β-lactamases. This indicates that the NDM-1 enzyme can hydrolyze cefiderocol but only can partially contribute to a reduced susceptibility to cefiderocol in *Acinetobacter*, consistent with Poirel’s research (42). There is a possibility that the prevalence rate of *bla*_NDM-1_ in *Acinetobacter* might be poised to increase since cefiderocol has been put in use.

### Conclusion

We characterized the phenotypic and genotypic features of five IMP-8/NDM-1-producing *Acinetobacter* spp. strains isolated in 2010. This research provides valuable insights into the development of antibiotic resistance in *Acinetobacter* species, emphasizing the importance of ongoing surveillance and genetic characterization in the context of emerging resistance patterns.

## Data Availability

The complete sequence of chromosomes and plasmids of all the strains in this study has been submitted to GenBank under accession numbers CP132915-CP132919 (A207), CP133054-CP133059 (A774), CP133052-CP133053 (A1014), and CP133047-CP133051 (A1269).

## SUPPLEMENTAL MATERIAL

Supplemental material spectrum.01158-24-s0001.pdf Table S1; Fig. S1 to S4.

ASM does not own the copyrights to Supplemental Material that may be linked to, or accessed through, an article. The authors have granted ASM a non-exclusive, world-wide license to publish the Supplemental Material files. Please contact the corresponding author directly for reuse.
