# Supplementary material for: Phenotypic and genotypic characterization of clinical carbapenem-resistant Acinetobacter species harboring the metallo-beta-lactamases IMP-8 or NDM-1 in China
Source: Microbiol Spectr. 2024 Dec 27;13(2):e01158-24. doi: 10.1128/spectrum.01158-24 (PMC11792499; doi:10.1128/spectrum.01158-24)
Supplement: Supplemental material — Table S1; Fig. S1 to S4. [file spectrum.01158-24-s0001.pdf]

Table S1. Primers used to confirm transconjugants.

	Forward primer	Reverse primer
IMP	TTTCCATAGCGACAGCACAGG	GTGATGCGTCCCCAATTTCAC
IMP-rep	CGGGCAAGCGTCATAAAGGA	CATCCAAGGCTACTCGCAAC
NDM	GCAAATGGAAACTGGCGACC	CAGCCACCAAAAGCGATGTC
NDM-rep	CCCGCCTAGAATGGGCTAAC	CAGCAACACGTACCGCATTT
ATCC17978_*fbsN*	CCTTTTGCAGTTGTGTAGAT	GCGAAAGTGAACAACGTA

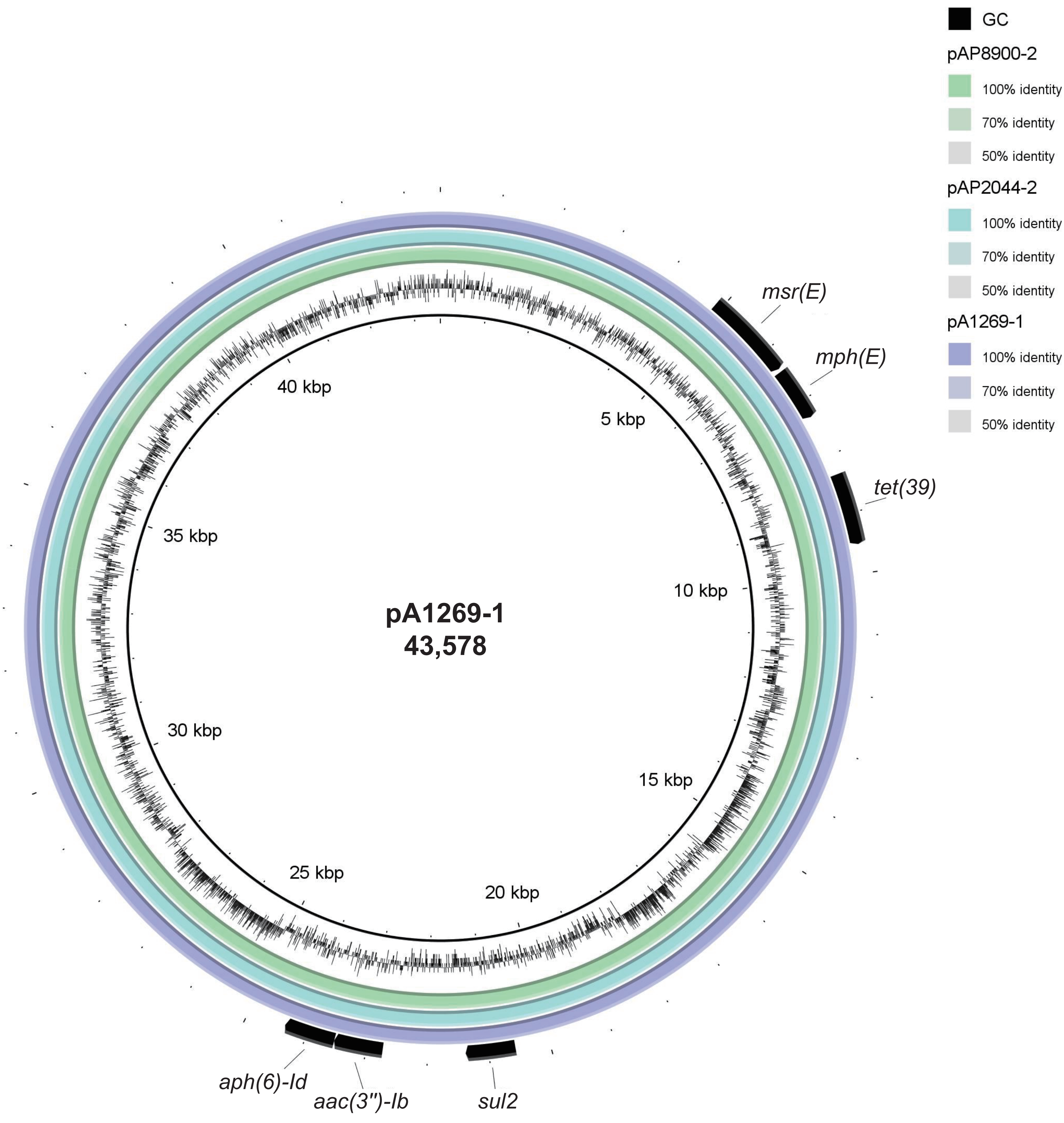

Figure S1 Circular map of GR22-type plasmid pA1269-1 in *A. pittii*.
Circular map of the pA1269-1 and comparison with pAP2044-2 (CP087718.1) and pAP8900-2 (CP123767.1) were completed using BRIG. Black ORFs represent resistance genes.

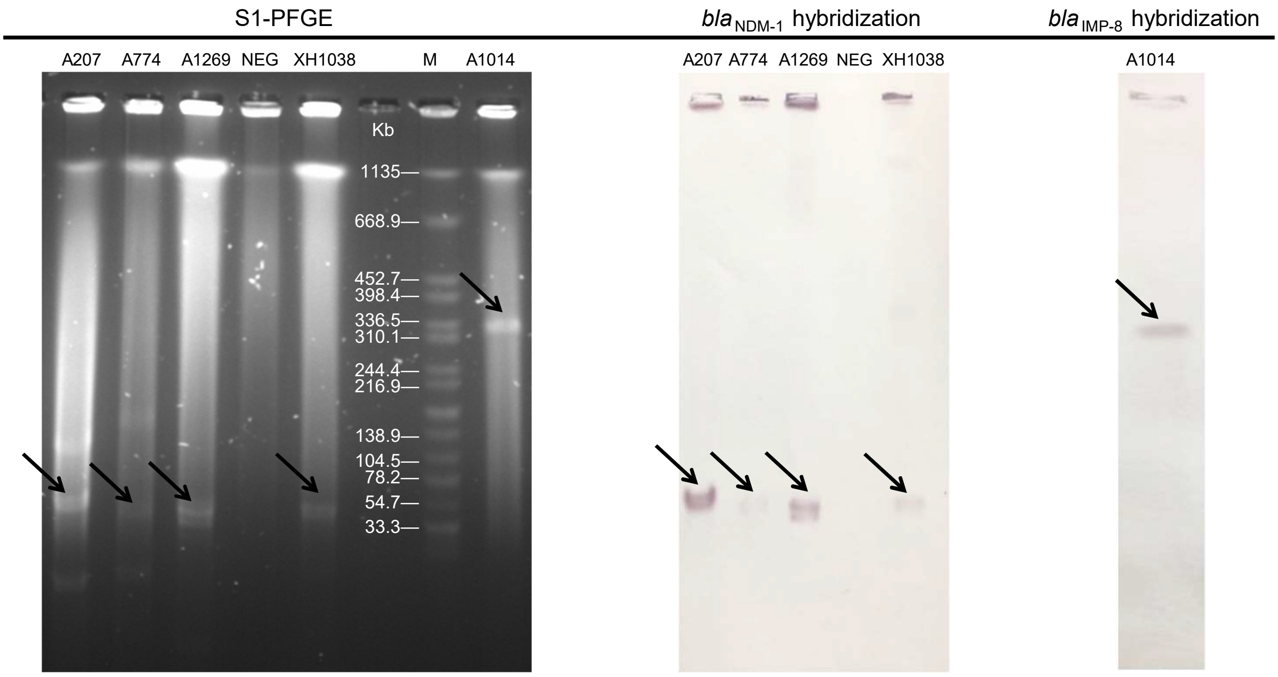

Figure S2. S1-digested plasmids and hybridization with *bla*$_{\mathbf{MBL}}$ of *Acinetobacter* isolates. M, marker, fragments of XbaI-digested genomic DNA from *Salmonella enterica* H9812. The bands with the black arrows represented positive signals of hybridization with NDM probe and IMP probe.

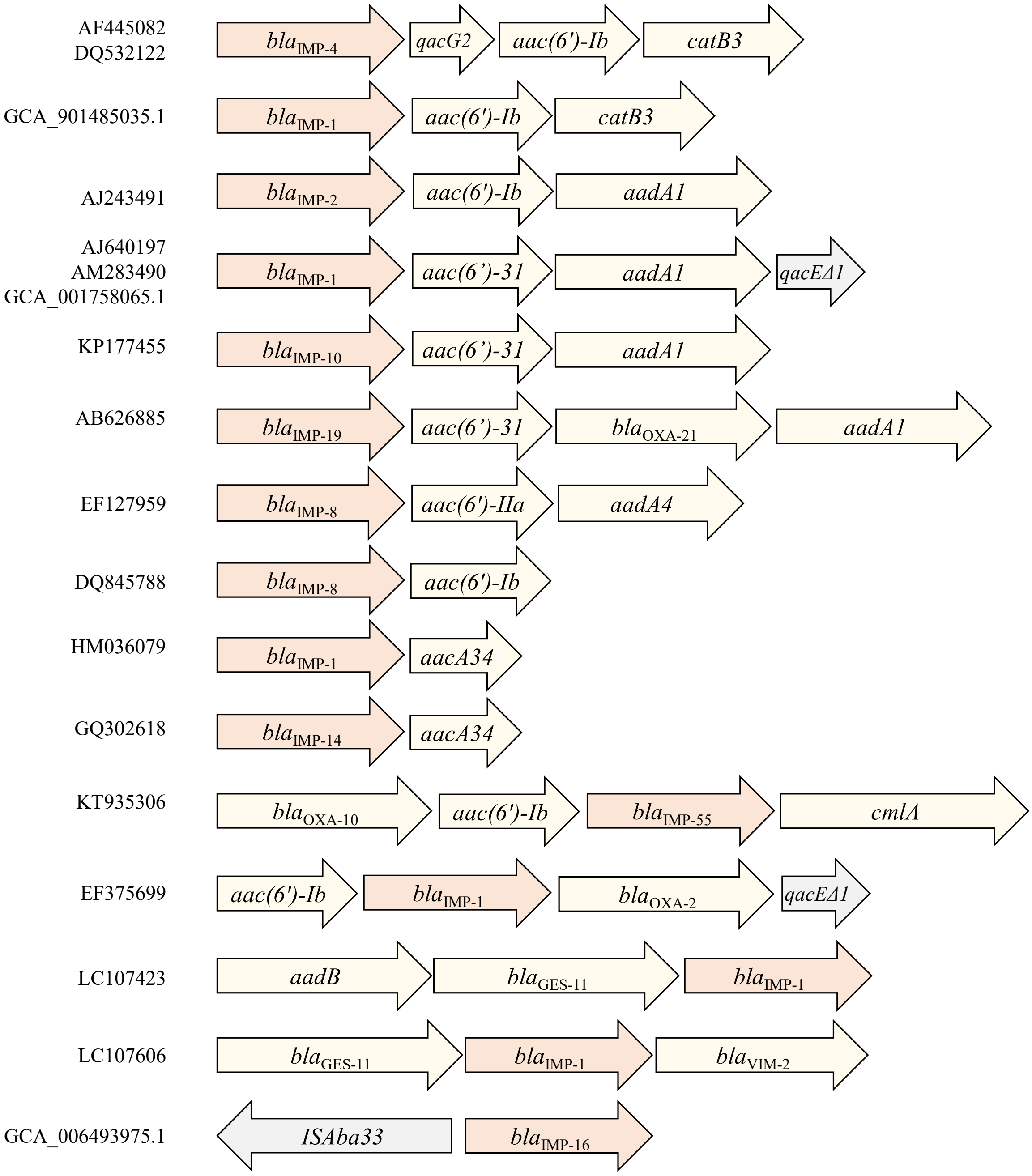

Figure S3. Comparison of incomplete integrons of *bla*$_{IMP}$-positive *A.baumannii* strains available at NCBI. Arrows indicated transcription directions of genes. The figure is not to scale.

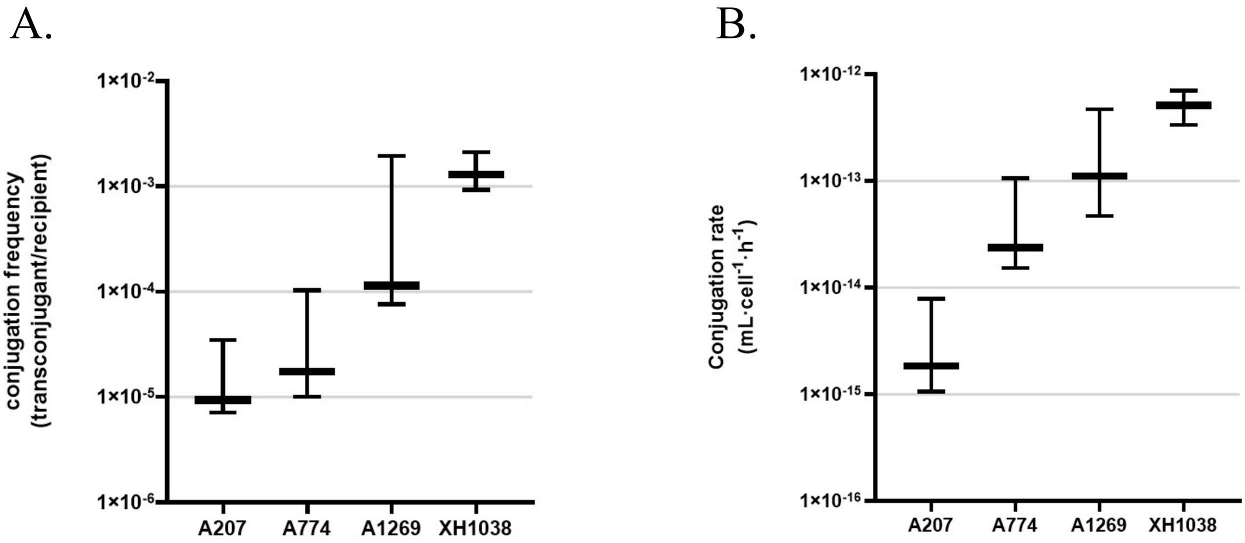

Figure S4. Conjugation frequency and conjugation rate.
Box and whisker diagram was used to show the range and the median.
